# A Virtual Reality Muscle–Computer Interface for Neurorehabilitation in Chronic Stroke: A Pilot Study

**DOI:** 10.3390/s20133754

**Published:** 2020-07-04

**Authors:** Octavio Marin-Pardo, Christopher M. Laine, Miranda Rennie, Kaori L. Ito, James Finley, Sook-Lei Liew

**Affiliations:** 1 Department of Biomedical Engineering, University of Southern California, Los Angeles, CA 90089, USA; marinpar@usc.edu (O.M.-P.); jmfinley@usc.edu (J.F.); 2 Chan Division of Occupational Science and Occupational Therapy, University of Southern California, Los Angeles, CA 90089, USA; christopher.laine@chan.usc.edu (C.M.L.); mrennie@usc.edu (M.R.); kaoriito@usc.edu (K.L.I.); 3 Division of Biokinesiology and Physical Therapy, University of Southern California, Los Angeles, CA 90089, USA

**Keywords:** biofeedback, stroke, brain–computer interface, neurorehabilitation, corticomuscular coherence, electromyography, co-contraction, virtual reality

## Abstract

Severe impairment of limb movement after stroke can be challenging to address in the chronic stage of stroke (e.g., greater than 6 months post stroke). Recent evidence suggests that physical therapy can still promote meaningful recovery after this stage, but the required high amount of therapy is difficult to deliver within the scope of standard clinical practice. Digital gaming technologies are now being combined with brain–computer interfaces to motivate engaging and frequent exercise and promote neural recovery. However, the complexity and expense of acquiring brain signals has held back widespread utilization of these rehabilitation systems. Furthermore, for people that have residual muscle activity, electromyography (EMG) might be a simpler and equally effective alternative. In this pilot study, we evaluate the feasibility and efficacy of an EMG-based variant of our REINVENT virtual reality (VR) neurofeedback rehabilitation system to increase volitional muscle activity while reducing unintended co-contractions. We recruited four participants in the chronic stage of stroke recovery, all with severely restricted active wrist movement. They completed seven 1-hour training sessions during which our head-mounted VR system reinforced activation of the wrist extensor muscles without flexor activation. Before and after training, participants underwent a battery of clinical and neuromuscular assessments. We found that training improved scores on standardized clinical assessments, equivalent to those previously reported for brain–computer interfaces. Additionally, training may have induced changes in corticospinal communication, as indexed by an increase in 12–30 Hz corticomuscular coherence and by an improved ability to maintain a constant level of wrist muscle activity. Our data support the feasibility of using muscle–computer interfaces in severe chronic stroke, as well as their potential to promote functional recovery and trigger neural plasticity.

## 1. Introduction

A growing body of evidence suggests that movement rehabilitation in the chronic phase of stroke (greater than 6 months post stroke) can be effective even for those with severe impairment, provided that the intensity and duration of therapy is much higher than what is commonly assessed in studies of rehabilitation [1,2,3,4]. Accordingly, there is a pressing need for automated or at-home training tools that can guide therapeutic practice while motivating the required high frequency of training [5]. However, for these systems to gain widespread application, it is critical to understand which movement-related signals are the most practical to monitor and effective to reinforce.

Brain–computer interfaces (BCIs) that trigger game activity upon detection of movement-related brain signals, measured via electroencephalography (EEG), have received much attention over the last two decades [6,7]. These systems do not require active, volitional movement and can often just be driven by the imagination of movement, allowing them to be of benefit to those whose severe deficits preclude direct reinforcement of overt movement by closing the loop between the brain and the environment [8,9]. However, it is known that not all individuals are able to learn how to modulate their brain activity, and much research is needed to predict who can control a BCI, even in the absence of a clinical condition [10]. Further, the practicalities of at-home, self-administered EEG remain a significant challenge [11,12].

An alternative bio-signal to reinforce is muscle activity, recorded through electromyography (EMG). Much effort has been dedicated to developing systems and protocols that use EMG to assist in stroke rehabilitation, for example, controlling exoskeletons [13,14] and providing feedback as a complement to traditional interventions [15]. Research has previously shown that even those with little or no active range of motion can often activate muscles, albeit weakly and with involuntary co-activation of antagonist muscles, which prevents the intended movement [16,17,18]. While EMG-based biofeedback training has often been reported to have positive effects [19,20,21,22,23], relatively few studies have attempted to use EMG feedback as a way to monitor and suppress unintended co-contractions [24,25], which could ultimately prevent gains in motor recovery even if muscle strength is increased. Thus, the goal of the current system is to examine the use of EMG feedback to specifically train individuals to reduce unintended co-contractions.

Recently, we tested the feasibility of a BCI rehabilitation system (REINVENT), which used EEG signals to trigger the movement of a realistic virtual arm within an immersive virtual reality (VR) environment [26]. The use of a realistic arm matches intent with outcome and may engage the purported action observation network, similar to mirror therapy [27,28,29]. The EEG version of the system [26] showed promising results in terms of user satisfaction and produced modest improvements in clinical assessments of deficits. However, for individuals who could activate their muscles, we noted the possibility that reinforcement of even trace muscle activity may have been as effective, if not more, compared with EEG.

Therefore, in the current pilot study, we tested the feasibility of an EMG-based variation of the REINVENT training system and explored training-related changes in clinical presentation and neuromuscular control. Specifically, we recruited four individuals in the chronic stage of recovery, who had less than 15 degrees of voluntary wrist extension and unintended flexor-extensor co-coactivation during attempted movement. They completed a series of seven 1-hour training sessions during which our system reinforced extensor activation without concurrent activation of flexors.

A battery of standardized clinical assessments was administered before and after training to monitor generalized improvement in motor function. In addition, before and after training, we probed changes in neural control of flexor and extensor muscles (separately) as participants attempted to hold a steady level of muscle activation using EMG feedback and a visual target. We quantified training related changes in task performance as well as in corticomuscular coherence, which measures synchronization between EEG and EMG oscillations. Corticomuscular coherence in the 12–30 Hz frequency range has been used to probe corticospinal tract integrity and neural recovery after stroke [30,31,32,33,34].

We hypothesized that that (1) reinforcement of EMG activity in participants with severe movement deficits would be feasible, safe, and provide a positive user experience for participants; (2) training would produce modest improvements in clinical assessments comparable to what we have previously observed for EEG-based neurofeedback (i.e., variable improvement across individuals, but with some participants showing clinically meaningful effects); and (3) that we would observe evidence of improved neuromuscular control as indexed by task performance and by enhanced beta band (12–30 Hz) corticomuscular coherence. Finally, we expected that participants who showed strong post-training changes in clinical assessments and neuromuscular control would also show large improvements in task performance during training, assuming that within-task performance is sensitive to training-induced neural plasticity.

## 2. Materials and Methods

### 2.1. Participants

We recruited four stroke survivors for this pilot study. Inclusion criteria required that each participant was in the chronic phase of recovery (>6 months since stroke onset); presented with upper extremity hemiparesis; was not taking anti-spasticity medication; and had no receptive aphasia, significant vision loss (corrected vision was acceptable), secondary neurological disease, or hand contractures. We specifically sought individuals with limited active wrist extension, as control over wrist extensor muscle activity was to be trained within our intervention. All participants gave written informed consent, and the protocol was approved by the Institutional Review Board of the University of Southern California (reference number: HS-17-00916, approved on 9/24/2019). Furthermore, none of the participants were receiving standard physical therapy and all participants that were part of other exercise programs agreed to pause such exercises for the duration of the study. We also assume that most spontaneous biological recovery had plateaued for all of our participants because they had their stroke at least 2 years prior to our intervention [35,36]. Participant characteristics are listed in Table 1.

### 2.2. Study Timeline

**Figure 1:**
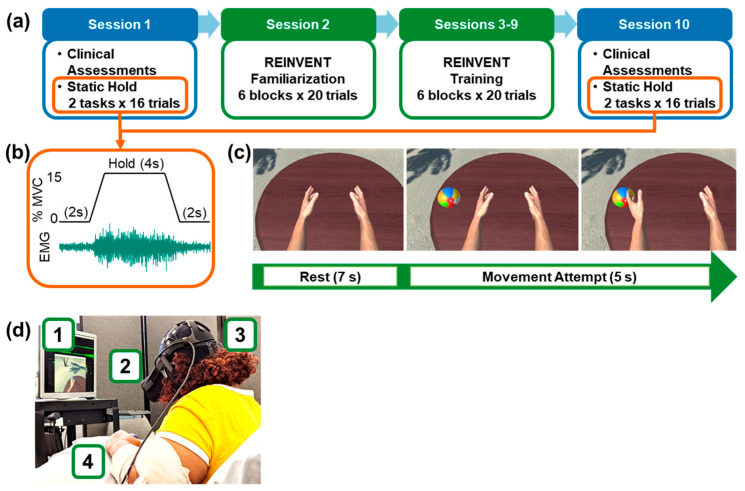
Experimental protocol. (**a**) Timeline of the 10-session program. Sessions 1 and 10 comprised a battery of standard clinical assessments and a test of neuromuscular control (static hold with electromyography (EMG) and electroencephalography (EEG) recording). Session 2 allowed for familiarization of the participants with the equipment and training paradigm. Our training program spanned seven sessions (session 3 through 9), focused on targeted wrist extensor activation of the more affected limb. (**b**) Muscle control test (static hold). In sessions 1 and 10, we tested control of wrist flexor and extensor muscles using a task in which participants were to maintain a constant level (15% maximal voluntary contraction (MVC)) of muscle activity for sixteen 4-second epochs using feedback of wrist muscle EMG and a target displayed on a computer screen. During this test, task performance and coherence between EEG and EMG signals were measured. (**c**) REINVENT training task. A single trial consisted of 7 s of rest and 5 s of attempted movement where, upon success, a virtual arm pushed a beach ball off a table. A successful trial required 2 s of extensor EMG activity that (1) exceeded 30% of the maximal activity as recorded during a power grip, and (2) was proportionally larger than unintended flexor activity, as determined by an extension ratio that adaptively increased or decreased depending on subject performance. A total of 120 trials were performed per session. (**d**) REINVENT system. Consisting of (1) acquisition and processing computer, (2) VR headset, and (3) EEG and (4) EMG sensors placed over the flexor carpi radialis (FCR), flexor carpi ulnaris (FCU), extensor carpi radialis (ECR), and extensor carpi ulnaris (ECU).

Each participant visited the lab for ten sessions (1–2 h each) over the course of two weeks. An outline of the study elements is shown in Figure 1, with each element detailed further below. Briefly, in sessions 1 and 10, we performed clinical assessments of upper limb function, grip strength, and wrist mobility (Figure 1a). We also performed an assessment of muscle control (static hold), in which participants used feedback of their wrist EMG amplitude from flexors and extensors (separately) to match a target level of activation for 16 trials of 4 s each (Figure 1b). During this test, we also recorded EEG over the ipsilesional and contralesional motor cortices to evaluate corticomuscular coherence. During session 2, participants were familiarized with our VR wrist-extensor training system. During sessions 3–9, the training intervention was provided for 1 h each.

### 2.3. Clinical Assessments (Sessions 1 and 10)

An occupational therapist performed clinical cognitive and motor assessments as part of the pre- and post-training evaluations. These assessments included the following:

- Fugl–Meyer assessment for the upper extremity (FMA-UE). This scale measures sensorimotor impairment of the upper limb following a hemiplegic stroke, including movement, coordination, and reflexes, and provides a score that ranges from 0 (greatest impairment) to 66 (least impairment) [37].
- Action research arm test (ARAT). This scale measures functional performance of the upper limb in terms of the ability to functionally manipulate objects with different sizes, weights, and shapes, and provides a score that ranges from 0 (greatest impairment) to 57 (least impairment) [38].
- Montreal cognitive assessment (MOCA). This is an assessment of cognitive impairments evaluating visuospatial abilities, memory, attention, concentration, language, and orientation, and provides a score that ranges from 0 (greatest impairment) to 30 (least impairment) [39].
- Sixteen-question stroke impact scale (SIS-16). This assessment consists of a series of self-reported questions evaluating quality of life as related to strength, hand function, mobility, and activities of daily living, and provides a total score that ranges from 16 (greatest impairment) to 80 (least impairment) [40].
- Wrist range of motion (ROM). Using a goniometer, we recorded the maximum degrees of passive and active wrist extension, wrist flexion, ulnar deviation, and radial deviation. Activities of daily life usually require 40 degrees of wrist extension, 40 degrees of wrist flexion, and 40 degrees of combined ulnar and radial deviation [41].

### 2.4. Additional Data Acquired

- Grip strength (GS). In each session, we recorded maximal grip force from the more affected hand using an analog dynamometer, while recording the associated EMG.
- Simulator sickness questionnaire (SSQ). In sessions 2 and 9, we evaluated each participant’s comfort with the VR environment using this 16-question survey covering oculomotor discomfort, disorientation, and nausea. The total score ranges from 0 (no sickness induced) to 63 (highest values of sickness) [42].
- Finally, we qualitatively evaluated the participants’ overall experience and feedback in terms of enjoyment and ease of use with a free-form questionnaire at the end of the experiment.

### 2.5. Physiological Recordings and Analysis

For all sessions, we measured surface EMG signals from four muscles at 2000 Hz using a Delsys Trigno Wireless System (Delsys Incorporated, Natick, USA). The Delsys Trigno EMG sensors were taped to the skin above the flexor carpi radialis (FCR), flexor carpi ulnaris (FCU), extensor carpi radialis longus (ECR), and extensor carpi ulnaris (ECU) of the more affected limb. The skin was cleaned with isopropyl alcohol and electrodes were positioned using double-sided tape and wrapped with a bandage. Proper positioning was confirmed via palpation and observation of EMG during attempted wrist extension, flexion, radial, and ulnar deviation, and light grip. These signals were down-sampled to 1000 Hz for offline storage and analysis.

Additionally, in the first and last sessions (1 and 10), we also recorded EEG at 500 Hz over the right and left motor cortices using a Starstim 8 System (Neuroelectrics, Barcelona, Spain). Electrodes were positioned at frontal-central (FC3, FC4), central (C3, C4, C5, C6), and central-parietal (CP3, CP4) scalp locations. This is the same system and electrode montage we used to provide neurofeedback in our previous study [26]. Here, we use EEG only to assess changes in corticospinal connectivity after EMG-based training. These signals were interpolated to 1000 Hz for offline storage and analysis.

### 2.6. Static Hold Task: Characterization of Muscle Control during EMG Amplitude Target Tracking (Sessions 1 and 10)

We sought to determine whether training influenced the degree to which participants could control their wrist muscle activity, as distinct from performance during the training task. Therefore, participants were asked to maintain a constant level of extensor EMG during attempted wrist extension and flexor EMG during wrist flexion. For each task, the flexor or extensor muscle with the largest signal to noise ratio during voluntary activation was chosen to provide EMG feedback. The chosen EMG signal was smoothed and rectified in a 1-second moving window to control the height of a feedback cursor that moved left to right across the computer screen for 10 s before looping back. The target participants were required to reach was the 4-second hold phase (plateau) of a trapezoid spanning 6 s (Figure 1b). The target’s hold phase was set to 15% of the tracked muscle’s maximal EMG, as established during the power grip. Two 90-second trials were completed for wrist extension and again for wrist flexion. At least 1 min of practice was provided for each task prior to recordings, and the first trial was removed so that all analyzed static holds were preceded by 4 seconds of rest. This resulted in 16 total holds per direction (flexion or extension), per participant. The order of flexion versus extension for each session was randomized. The flexion trials were executed with the hand in a supine position and the extension trials were executed with the hand in the prone position. The task is quasi-isometric for these participants as the required level of muscle contraction resulted in little if any overt movement of the wrist. Participants were asked to rest their more affected arm on a pillow, and they were provided with a low-stiffness stress-relief ball to keep their fingers from involuntarily curling uncomfortably as they attempted the task. We discouraged participants from actively attempting to grip the ball.

*Error quantification*: We quantified the accuracy with which participants could maintain a stable level of muscle activation by calculating the median absolute deviation of the feedback cursor from the target during the last 3 s of each hold phase.

*Corticomuscular coherence*: Along with calculation of tracking error, we measured synchronization between EEG and EMG signals during the same time epochs. This measure, called corticomuscular coherence, is a frequency-domain correlation where a value of 0 marks no correlation between signals at a given frequency, and 1 indicates perfect correlation. We were particularly interested in evaluating any changes in 12–30 Hz range (e.g., beta band) neural drive to muscles, as this is commonly detected during static muscle contractions and used to probe the integrity of corticospinal communication [30,33,34,43,44,45,46,47,48].

EEG signals were first bandpass filtered between 5 and 100 Hz using a sixth order, zero-phase Butterworth filter, and re-referenced to the common average after removing any noisy or bad channels, which were identified via manual inspection and using an artifact reconstruction method within Matlab’s EEGLAB toolbox [49,50]. Channels C3 and C4 were evaluated for coherence with wrist EMG signals. If one of these channels had to be excluded, FC3 and FC4 were used instead. The choice is unlikely to have influenced our results, as these electrodes are close to each other, and previous studies have shown that corticomuscular coherence is not precisely localized (even to the contralateral hemisphere) during unimanual actions in this study population [31,33]. EMG signals for each epoch were bandpass filtered between 15 and 450 Hz, the amplitude envelope extracted using the Hilbert transform [51,52,53], and the resulting signals were normalized to have zero-mean and unit variance [54,55]. For each task, pooled coherence [56] was calculated between the EEG (both ipsilesional and contralesional) and EMG signals from the two active extensors or flexors. Trial epochs were first concatenated, and then coherence was calculated using the *mscohere* function in Matlab, specifying 512 ms Hann-windowed segments with 75% overlap. A 95% confidence level for each coherence profile was calculated using Equation (1) [57,58]:

(1) $$CL=1-0.05^{\frac{1}{L-1}\;},$$

where *L* is the number of segments used to calculate coherence, adjusted for tapering, and overlap as in [59]. For a group-level analysis, we repeated the above procedure after concatenating data from all four participants. Using this technique, the same statistical methods can be used to evaluate group-level data as used for individual coherence profiles [56].

### 2.7. Wrist Extensor Training in Virtual Reality (Sessions 2–9)

We utilized the same task in sessions 2 through 9; however, we excluded session 2 from our analyses as it was a familiarization session during which participants were allowed to ask questions during the task. Our training paradigm (sessions 3–9) utilized an Oculus Rift CV1 (Facebook Technologies, Menlo Park, USA) head-mounted display system with 3D-audio headphones. We used the lab streaming layer (LSL) protocol [60] for data synchronization between acquisition and feedback systems. The VR task was programmed in the Unity game engine (v2017.4.1, Unity Technologies, San Francisco, USA) and rendered with the Oculus SDK, as per our previous work [61]. EMG signals were processed and analyzed online with custom scripts in Matlab (R2014a, The Mathworks, Natick, USA).

Participants were shown a visualization of their two arms resting on a table, as shown in Figure 1c. The virtual arms were chosen from a set of models to best match each individual’s physical characteristics. Each trial consisted of 7 s of rest and a 5-second movement attempt window during which participants were required to maintain a threshold of EMG activation for 2 s for the virtual hand to push the ball off the table. EMG feedback was given in real-time during the movement attempt window, such that a decrease in the EMG signal led to the hand moving towards the start position and an increase led to the hand moving towards the end goal. This was repeated for six blocks of 20 trials for each session, lasting approximately 1 hour. In this training paradigm, we sought to reinforce wrist extensor activation without simultaneous flexor activation. As mentioned previously, this is advantageous over simple EMG activation as a feedback signal because unintended co-contraction of antagonists may be just as, if not more, detrimental to voluntary movement than total paralysis [18,62], and we found this to be the case with our study participants as well. We thus calculated an extensor ratio (ER), shown in Equation (2):

(2) $$ER=\frac{EMG_{extensors}}{EMG_{extensors}+EMG_{flexors}},$$

as the sum of the extensor activity divided by the sum of the extensors and flexors. This calculation was made at 250 Hz, using a 480 ms moving window of EMG. Each signal was rectified, averaged, and normalized to the maximal activity recorded during a maximal power grip. For a successful trial, two thresholds had to be exceeded. First, the summed extensor activity was required to exceed 30% of its maximal level during the power grip, and second, an adaptive ER threshold had to be exceeded. The ER threshold was initially set to 0.5 (equal flexor and extensor activity) at the beginning of each session and would increase or decrease in increments of 0.3 (within the range of 0.3 to 0.97) if the previous three trials were all successes or failures, respectively. This was implemented to comply with current recommendations that training tasks should be challenging and progress in difficulty to encourage continuous and adaptive learning [63]. Participants were to rest their more affected arm on a pillow, and they were provided with a low-stiffness stress-relief ball to keep their fingers from involuntarily curling uncomfortably as they attempted the task. We discouraged participants from actively attempting to grip the ball. Importantly, such a strategy would lead to task failure because this grip would result in co-contraction of flexors and extensors, leading to a low ER.

### 2.8. Statistical Analyses 

We utilized custom scripts in Matlab (R2019a, The Mathworks, Natick, USA) and R (R Foundation for Statistical Computing, Vienna, Austria) for offline signal processing and statistical analyses, respectively. 

#### 2.8.1. Behavioral and Neuromuscular Changes Following Training

*Clinical assessments*: We performed paired *t*-tests (N = 4) to identify the strongest and most consistent changes in FMA-UE, ARAT, SIS-16, SSQ, ROM, and grip strength. These group-level tests were considered separate, and thus significant at the *p* < 0.05 level without correction. In addition, owing to the small number of subjects, we also report non-significant trends (*p* < 0.1) as an exploratory analysis.

*Static hold (EMG tracking performance)*: The mean and standard deviation of tracking error were calculated across the 16 attempts for each participant before and after the training intervention. Group-level effects were identified using a paired *t*-test to compare pre- versus post-training error across individuals. Additionally, a paired *t*-test was used to evaluate the significance of any training-induced changes in performance at the individual level. The significance level for each individual was set to *p* < 0.05.

*Corticomuscular coherence during tracking*: We evaluated changes in corticomuscular coherence at the group level, with a Z-score difference of coherence [58] (pre versus post) at each frequency of the group-level coherence profiles constructed for each task (flexion or extension) and hemisphere (ipsilesional and contralesional) using the formula given in Equation (3):

(3) $$Z_{diff}=\frac{FZ_{post}-FZ_{pre}}{\sqrt{1/L}},$$

where *FZ* is the Fisher-transformed coherence value (i.e., atanh (sqrt (Coh)) and *L* represents the degrees of freedom, calculated as described for Equation (1). This provides a standard Z score for the difference in coherence between sessions 1 and 10, for every frequency. Then, we created a composite Z-score for the 12–30 Hz beta band using Stouffer’s Z-score method [64]. We chose the lower bound (12 Hz) for better comparability with a previous study [31], in which beta band corticomuscular coherence was found to differ according to recovery status. Composite Z-scores with an absolute value above 1.96 are considered significant at the 5% confidence level. For completeness, two other bands, alpha (8–12) and gamma (30–50), were tested as well.

#### 2.8.2. Changes across Training Sessions

*Performance*: We calculated the average within-game ER-threshold and % successful trials to track changes in task performance over training sessions for each participant. A Spearman’s rank correlation was used to determine if these measures increased or decreased consistently across days, and paired *t*-tests of the first versus last session were used to test for any consistent group-level effects.

*EMG during training*: The same procedure was utilized for three metrics of EMG activity (flexor amplitude, extensor amplitude, and ER). For the analysis of flexor and extensor amplitude changes over sessions, we normalized the mean rectified voltage for each EMG signal by the mean and standard deviation of those recorded in session 3 (Z-score). This normalization allows for better comparability between participants. Although the method is susceptible to small variations in electrode placement and signal quality across sessions, it avoids dependence on EMG activity during maximal voluntary effort. Furthermore, for our study population, this maximal voluntary effort does not represent the full capacity of the muscles, and thus cannot be assumed to be stable across sessions. We assessed changes over time as before, using Spearman’s rank correlation for individuals, and a paired *t*-test for group-level change.

*EMG during maximal power grip*: Finally, we assessed grip strength and EMG activity recorded during the daily maximal power grips. We calculated the amplitude of each EMG signal as well as the ER at maximal grip force. Similarly, we assessed changes over time using Spearman’s rank correlation for individuals and a paired *t*-test for group-level change.

## 3. Results

### 3.1. Feasibility

Participants reported minor levels of discomfort after training with the head-mounted display, assessed with the simulator sickness questionnaire, during both the first and last training sessions (first: mean = 5.56, SD = 5.27; last: mean = 6.35, SD = 2.59). Changes in such discomfort were not significant when comparing both sessions (*t* = 0.32, *p* = 0.76). Qualitatively, all participants liked the virtual environment and commented on enjoying the experience. They also reported using different strategies to improve their control on the task such as concentrating on different parts of their hands, imagining the movement, or focusing on muscle sensations. All reported that they would be enthusiastic to use a portable home system if one were available. Although wrist mobility was severely limited in all participants, all participants were able to activate their muscles enough to generate detectable activity during EMG-based training and assessments. This is particularly relevant because participants with this level of severity would often be considered primarily suitable for EEG-based neurofeedback under the assumption that no direct reinforceable motor commands exist.

### 3.2. Behavioral Changes Following Training

**Figure 2:**
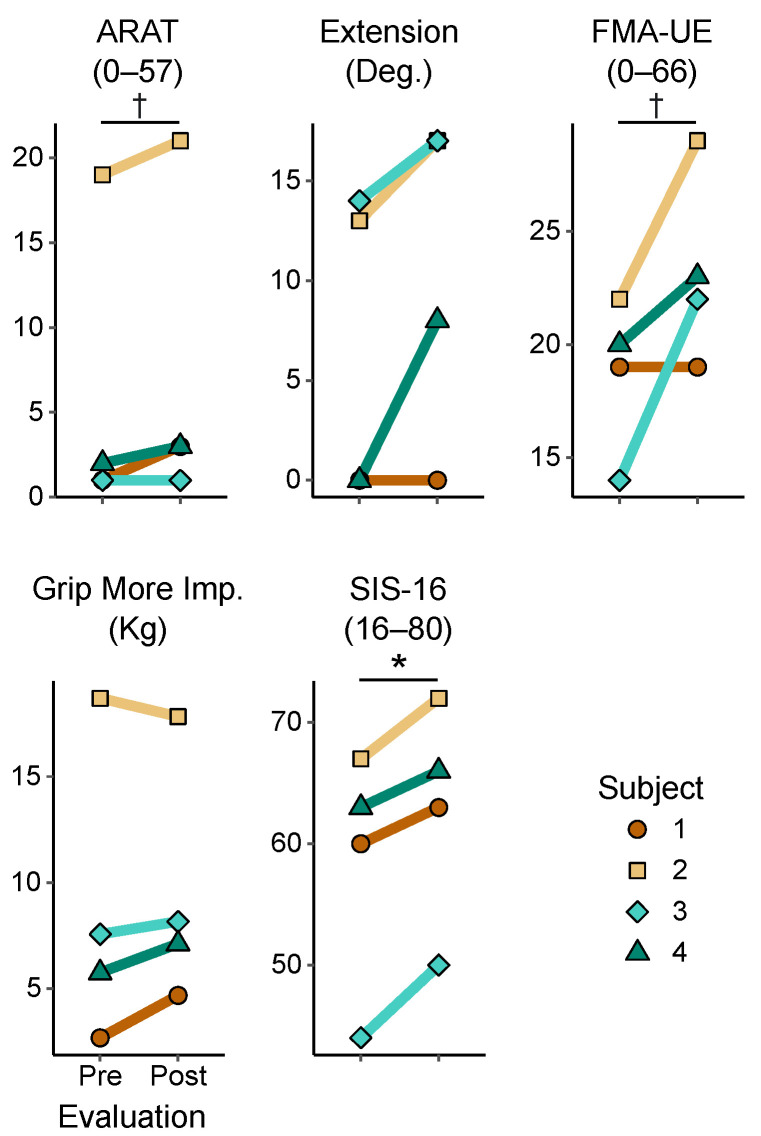
Clinical assessments. Assessments for each participant, as evaluated in sessions 1 and 10. * indicates group-level significance of *p* < 0.05. † indicates a group-level nonsignificant trend at *p* < 0.1. FMA-UE, Fugl–Meyer assessment for the upper extremity; ARAT, action research arm test; SIS, stroke impact scale.

At the group level, only the SIS-16 showed significant improvements (*t* = 5.67, *p* = 0.011; Figure 2). We found non-significant trends in that ARAT (*t* = 2.61, *p* = 0.079) and FMA-UE (*t* = 2.43, *p* = 0.093). The range of active wrist extension improved for three participants, but the effect was quite variable across individuals (*t* = 2.27, *p* = 0.108). In addition, three participants that improved their FMA score, with two (participants 2 and 3) showing an improvement meeting the minimal clinically important difference (MCID) criteria of 4.25–7.25 points [65]. None of the other measures showed significant group level changes, as shown in Table 2.

### 3.3. Changes of Muscle Control during EMG Amplitude Target Tracking

**Figure 3:**
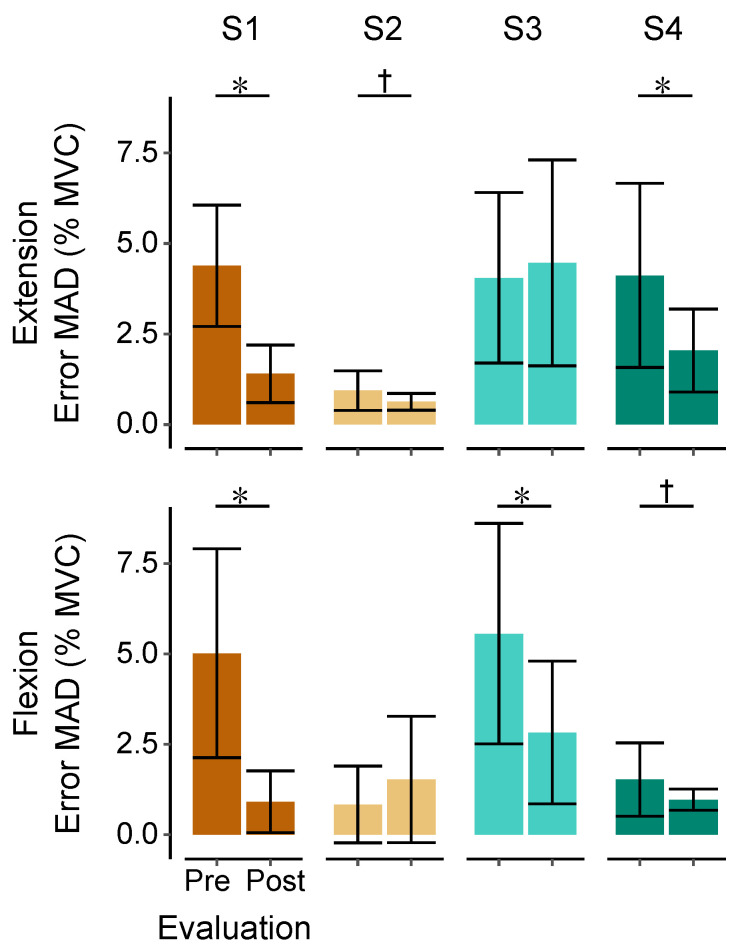
Performance changes in EMG amplitude control. Individual changes (1–4, left to right) in tracking performance (median absolute deviation (MAD) error) during maintenance of a constant level of extensor (top) or flexor (bottom) activity before and after seven sessions of wrist extensor training. Note that the training itself did not require maintenance of a constant level of EMG, nor was direct EMG feedback provided. Improvements were seen in both tasks after training. * indicates a significant change at the 95% confidence level. † indicates a nonsignificant trend at *p* < 0.1. Bar heights display the mean error and error bars display +/−1 standard deviation.

Trends of improved motor control, as measured by a reduction in the median absolute deviation from the target, were seen in three of four participants after training, for both flexion and extension tasks (extension: *t* = −5.69, *p* < 0.001; *t* = −2.04, *p* = 0.06; and *t* = −3.60, *p* = 0.003 for participants 1, 2, and 4, respectively; flexion: *t* = −5.09, *p* < 0.001; *t* = −2.67, *p* = 0.018; and *t* = −2.02, *p* = 0.061 for participants 1, 3, and 4, respectively). All three statistically significant individual tests (*p* < 0.05) were also significant at the Bonferroni-corrected 95% confidence level for eight tests (*p* < 0.0063), which argues against the possibility that these results are the incidental outcome of making eight statistical tests. However, a paired *t*-test at the group level showed nonsignificant *p*-values of 0.21 and 0.22 for extension and flexion tracking error, respectively. Figure 3 shows the tracking error before and after training for each participant.

### 3.4. Neuromuscular Changes Following Training

**Figure 4:**
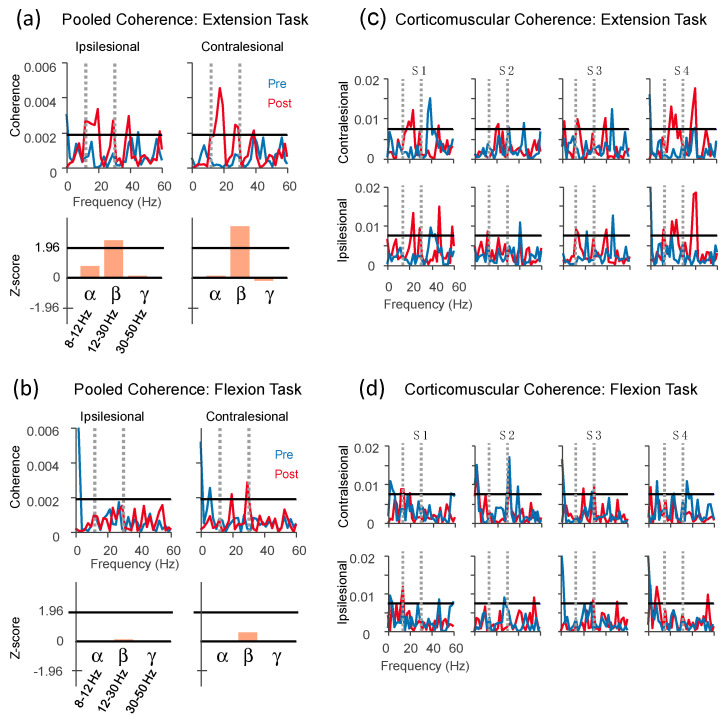
Corticomuscular coherence during static flexion and extension. (**a**) Group-level pooled coherence of ipsilesional and contralesional EEG to wrist extensor muscles during extension task. Horizontal lines indicate the 95% confidence level. Bar plots below the spectra represent the composite difference of coherence before vs. after training within three frequency bands. Beta-band coherence was increased significantly and bilaterally after training. (**b**) Coherence during the flexion task was generally not present either before or after training. Panels (**c**) and (**d**) show the individual coherence spectra for each participant (1–4, left to right). Coherence within 0 and 60 Hz is shown in all plots, including gray vertical dashed lines indicating the beta band (12–30 Hz).

Consistent, significant corticomuscular coherence was observed only during static holding of wrist extension and not during flexion. Pooled coherence across participants shows that, during maintained wrist extension, the only significant coherence occurred within the beta band (12–30 Hz) and only after training (Figure 4a, top, ipsilesional: Z-score pre–post difference = 2.57, *p* = 0.010; contralesional: Z-score pre–post difference = 3.29, *p* = 0.001). After training, the composite difference of coherence, that is, the averaged coherence within each frequency band, was evaluated (Figure 4a bottom, horizontal lines at Z-score = 1.96 indicate the threshold for significance at *p* < 0.05 level), and showed a significant effect of training only for the beta band. This change in beta-band corticomuscular coherence was significant at the group level for both ipsilesional and contralesional EEG. It was also specific to the wrist extension task; there was little or no coherence between EEG measured from either hemisphere and flexor EMG during the flexion task (Figure 4b). Individual coherence profiles showed similar effects, with significant peaks in the beta band in bilateral hemispheres post training for wrist extension, and no consistent effects for other conditions (Figure 4c,d). The magnitude and precise frequencies were variable, but it is clear that the group-level effects could not have occurred without consistency across subjects. For example, subject 4 showed a large peak in the gamma band (around 40 Hz) in the post-training contralesional recordings (Figure 4c), and yet this is not reflected in the pooled coherence (Figure 4a).

### 3.5. Changes across Training Sessions

**Figure 5:**
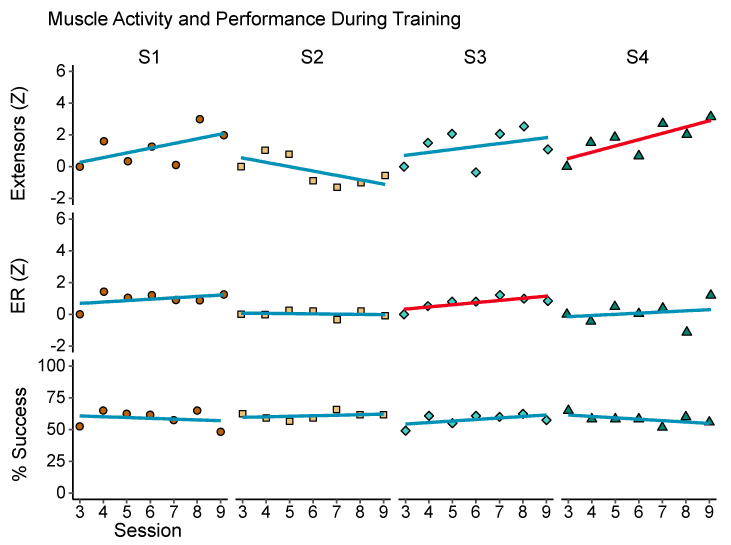
Muscle activity and performance during training. Each of the four columns represents the per-session average of one measure (rows) recorded during training. Top row: extensor EMG activity, normalized to the first session (Z-score). Middle row: extensor ratio (ER), or ratio of extensor activation relative to total muscle activity, also normalized to the first session. Bottom row: game performance as the percentage of trials in which participants exceeded the ER threshold, as well as a minimal activation threshold set to 30% of maximal wrist extension. Best fit lines are included to visualize trends across sessions. Red lines indicate that a Spearman rank correlation calculated between session number and the measure of interest, for that participant, exceeded the 95% confidence threshold.

At a group level, we found no significant changes in game performance or muscle activity during training, although the ER did show a non-significant trend (*t* = 2.58, *p* = 0.08). Table 3 contains group-level data for training-related muscle activity, game performance, and grip strength. At the individual level (Figure 5), measures of muscle activity were variable, but three of four participants showed increasing extensor activity over time, significant for participant 4 across sessions (Spearman’s rho = 0.86, *p* = 0.024). Similarly, participant 3 showed a significant increase in ER (rho = 0.86, *p* = 0.024). However, no consistent or significant improvements were found in the proportion of successful trials or increases in the ER threshold.

## 4. Discussion

### 4.1. Summary

In this pilot study, we explored the use of EMG feedback within a VR-based rehabilitation program targeting individuals with chronic, severe movement deficits from stroke. We found that seven 1-hour training sessions in which participants attempted to activate wrist extensor muscles without coactivation of flexors was both feasible in this study population and provided an acceptable overall user experience, with minimal discomfort stemming from the VR environment or the required task. Despite the relative brevity of the training program, we observed notable improvements in standard clinical assessments, the strongest being an improvement in the SIS-16 quality of life measure. We also saw non-significant trends towards improvements in the FMA-UE and the ARAT. Notably, as observed in previous studies, these improvements were highly variable across individuals, but they generally matched or exceeded what has been found using EEG-based neurofeedback. In addition, after training, participants significantly improved their ability to maintain a constant level of wrist flexor and extensor activation and, importantly, showed enhanced 12–30 Hz corticomuscular coherence, which we interpret as evidence of neural reorganization associated with functional recovery. Participants did not improve their performance during the training task itself, suggesting that post-training improvements in our various assessments were not owing to learned, task-specific behaviors, but instead, a more general influence of movement training on neural recovery.

### 4.2. Feasibility and Acceptability

Our first hypothesis was that an EMG-based version of the REINVENT system would be feasible and acceptable to users. Although we assumed that EMG would be detectable in anyone without severe flaccid paralysis, the utilization of very weak EMG within an entirely EMG-based training program has not been commonly reported for our targeted study population, leaving the feasibility of this approach unclear. One pilot study [25] and a later follow-up [24] demonstrated that a muscle–computer interface could be used to uncouple pairs of shoulder/elbow muscles. However, our task focused specifically on promoting individuated wrist extensor activity in participants with little to no functional wrist movement. Accordingly, our task may have been more difficult and would have produced weaker EMG signals.

We were able to detect useable EMG signals in all participants and confirmed that it was feasible to use these signals to drive our training paradigm. However, the use of EMG biofeedback in participants with such severe motor deficits has several important caveats that merit attention. First, raw EMG voltage varies across individuals and recording methods, and thus EMG amplitudes are typically normalized to a maximal level of activation determined for each individual. Our target population could not maximally activate their muscles to provide an unambiguous normalization. Furthermore, none of our participants could produce a muscle-specific ‘maximal effort’; as such, efforts always produced some degree of co-activation across all forearm muscles. We thus normalized our EMG using a power grip, which produces a consistent co-activation of all muscles, and thus reduced the dependence of our training system on the use of precise units of EMG amplitudes. This method also mitigates potential problems stemming from electrical cross-talk between nearby active muscles, as well as the complex association between EMG amplitude and voluntary effort, that is, when the raw EMG signal is dominated by the recruitment or de-recruitment of a small number of motor unit action potentials, as can be the case at very low levels of activation.

Moreover, we implemented an adaptive threshold to define success within our training paradigm (the extensor ratio, ER). This method provides an adaptive level of challenge, which is desirable for any personalizable rehabilitation program. However, as with any adaptive threshold, there is a need to balance delivery of progressive challenge with the practical requirement of maintaining participant engagement and motivation. This can be difficult to predict a priori. We found that all of our participants remained engaged with the task, and despite average success rates of just over 50% per session, none reported that the task was too difficult or frustrating. Anecdotally, most considered each session akin to a workout and felt it natural that the system would alter difficulty contingent on performance. Simulator sickness and general discomfort with using the VR headset were minimal, and most suggestions for improvement were directed at the monotony of the task, or specific preferences regarding the visual environment.

Overall, participants rated the training experience as positive, and despite several potential difficulties related to the use of EMG to control the virtual arm, our EMG-based variant of REINVENT was found to be feasible in all participants.

### 4.3. Clinical Assessments

We had initially hypothesized that, after training, we would see improvement in clinical metrics at least on par with what we had found in our EEG-based REINVENT training program [26]. This was partly based on our previous finding that three of four participants might have been more successful had EMG been used to trigger movement of the virtual arm rather than EEG. In that study, group-level effects were not statistically significant, but three of four participants showed improved FMA-UE scores, and one improved by 6 points, which meets the criteria for a clinically meaningful change (4.25–7.25 points [65]). In the present study, overall improvements may have been even stronger, with a significant group-level effect for the SIS-16 and positive, although nonsignificant, trends (*p* < 0.1) exhibited for ARAT and FMA-UE. For the FMA-UE, we again found improvement in three of four participants, but now two showed clinically meaningful differences (7 and 8 points). In fact, on average, there was a 4.5 point improvement in FMA-UE, which even compares well to longer training interventions [6].

Mugler et. al. found that six sessions of EMG-based training to reduce co-contraction was able to improve FMA-UE scores of 32 moderate-to-severe stroke survivors by an average of about 3 points [24]. This is the closest in design to the current study and confirms that this type of training can be of benefit in a larger population. While there is no direct EEG-only paradigm for training co-contraction, it is worth noting that many of these studies involve much higher doses. For example, the study by Bundy et. al. used 37 to 72 sessions to improve ARAT scores by 10 points on average [66]. Likewise, our training paradigm may benefit from continued use beyond seven sessions.

Because our training task was specific to wrist extension, improvements in ARAT, FMA-UE, or SIS-16 may indicate a broad, generalized effect of training. That is not to say there were no specific effects on the wrist muscles, as there was improvement in the range of active wrist extension for several individuals, but it is certain that these changes in wrist function do not explain changes in clinical metrics. Concerning range of motion, it should be noted that the standard test of active extension begins from a neutral wrist angle, and thus small gains in muscle strength or individuation may have been more apparent had we tested the ability to lift the wrist from a relaxed/hanging position, where the general tone of antagonist flexor muscles may have been lower at the initiation of extensor activation.

### 4.4. Neuromuscular Control

In addition to measuring training-induced changes in standard clinical assessments, we included a test intended to determine whether wrist extensor training within the REINVENT system produced generalizable changes in the neural control of either wrist extensor or flexor muscles. To disambiguate general effects from task-specific ones, we performed our analysis of neuromuscular control using a task that required fundamentally different behavior compared with our training paradigm. Specifically, we asked participants to maintain steady activation of a wrist extensor or flexor muscle given feedback of their EMG amplitude and a target. This allowed two separate (but related [67]) evaluations of neural control, one focused on motor function and the other on neural plasticity.

Our functional assessment was simply task performance. We had initially expected improvement after training, and indeed, three of four participants became more capable of maintaining a steady level of either flexor or extensor activity, as indexed by the median absolute deviation of their feedback cursor from the target. Because we found improved performance for both flexors and extensors, we interpret this finding to imply that training had a generalizable influence on voluntary control of muscles.

Given that neurorehabilitation is intended to produce useful changes in neural function, we also assessed corticomuscular coherence during the same static holding task. Corticomuscular coherence quantifies synchrony between EEG and EMG activity in the frequency domain, and high coherence is interpreted as a clear indication of functional communication and connectivity between the cortex and the motor neurons that innervate a given muscle [32,68,69,70]. It is worth noting that previous literature has shown inter-subject variability when measuring corticomuscular coherence, even in the absence of a clinical condition [71]. Nevertheless, corticomuscular coherence in the beta band (12–30 Hz) has often been used to probe the functional integrity of corticospinal communication following stroke [30,31,33,34,44,46,48,72,73]. Initially after stroke, corticomuscular coherence is reduced [44,46,73], but increases either with natural recovery [30,31,33] or recovery due to specific rehabilitation efforts [34,48]. Furthermore, changes in beta-band corticomuscular coherence correlate positively with corticospinal excitability and inversely with gamma-aminobutyric acid (GABA) mediated cortical inhibition [32,74,75,76]. For stroke survivors in the chronic stage of recovery, the location of maximal EEG–EMG coherence on the scalp is not necessarily over the primary motor cortex contralateral to the active limb, but instead, can be located over a broad area including bilateral supplementary and premotor areas, and may even be strongest on the contralesional hemisphere during activation of muscles on the more affected limb [31,33,77].

Our findings strongly suggest that training did impact corticospinal communication in our participants. First, we found that training produced frequency-specific effects on corticomuscular coherence. Only the beta band showed consistent, significant coherence across participants, and only after training. The change in coherence between session 1 and 10 was even statistically significant at the group level, suggesting that the post-stroke recovery of corticomuscular coherence [31,33,34] can be induced, even at the chronic phase, by a short-term behavioral intervention. Further, our data emphasize that rehabilitation paradigms targeting neural plasticity do not necessarily require detection or reinforcement of movement-related EEG oscillations. EMG may provide a sufficient (and natural) index of motor circuit operation when it can be detected.

Interestingly, training-induced enhancement of corticomuscular coherence was only observed when participants were asked to maintain wrist extensor activation. There were no changes in coherence (and more generally, no coherence) during the flexion task. This may relate to the fact that the trained task was designed to produce voluntary control over the extensors (not the flexors), and/or because wrist extensors may receive a greater proportion of monosynaptic corticospinal projections compared with the wrist flexor muscles [78,79]. Because changes in reticulospinal pathways may ultimately cause pathological synergistic muscle activation after stroke [80,81,82], it may be that there is a greater contribution of reticulospinal drive to wrist flexion compared with extension movements [83]. Accordingly, if one aim of our training task was to reduce unintended flexor activation, then the reinforced neural activity might have been a reduction of brainstem or reticulospinal output to the flexors. At the same time, our task may have positively reinforced direct corticospinal activation of the extensors, which, unlike an alteration in brainstem output, would have been reflected as a change in corticomuscular coherence.

### 4.5. Training Effects versus Task Performance

We had initially expected to find some association between task performance and the extent of improvement measured by our post-training assessments. This was premised on the assumption that task success might itself promote recovery, and further, that neural recovery induced by a particular task should improve performance of that specific task. Neither assumption is supported by our results. We found clear evidence of training-induced changes in clinical metrics and indices of neural recovery that would not be expected to have occurred spontaneously in this population, or by virtue of a given assessment having been previously tested two weeks earlier. At the same time, we found no consistent improvement in the performance of the training task across seven sessions. The training did, however, apparently promote some form of generalized recovery. Potentially, the training-induced changes that we observed reflect the early stages of a recovery process that would only later gain the strength to generate meaningful improvements in task performance. For example, improved utilization of the corticospinal tract may have occurred thanks to the constant movement attempts, and led to modest improvements in certain clinical assessments. However, given that our task training also demanded inhibition of involuntary flexor activity, this may have required a different (e.g., reticulospinal) circuit that did not, or had not yet, responded to training. Overall, our results emphasize that performance improvement within a rehabilitation paradigm is not necessarily a prerequisite of training-induced neural plasticity, nor potential improvements in clinical function.

### 4.6. Limitations and Conclusions

As with any pilot study, it is important to emphasize that our sample size does not allow us to generalize our findings to the larger population of individuals suffering from movement deficits due to stroke. Our results, while promising, must be interpreted with the understanding that both symptom expression and the progression of recovery can be highly variable across individuals. Even so, our results do suggest that EMG-based rehabilitation using the REINVENT system is feasible, and at least in some cases, can promote measurable improvements in clinical metrics and measures of neuromuscular control. Further, our findings emphasize that changes in neural activity, clinical outcomes, and performance of a training task are likely to occur on different time scales and to different degrees for different individuals. Understanding the relationships between these metrics will be critical for uncovering the mechanisms of neural recovery and optimizing rehabilitation protocols; however, studies with a larger sample size are required. Furthermore, EMG has potential for many extensions and variations that could provide more realistic interactions, like predicting body movements and their related forces and torques [14,84]. However, more research is required before introducing these mechanisms because, for example, the assumed linear relationships between EMG and force are lost after a stroke [85]. Importantly, another area worth exploring would be merging different feedback modalities, similar to the works of Kawase et al. [86] and Leeb et al. [87]. Although our present study focuses on exclusive utilization of EMG for reasons of practicality, it would be of utmost interest to investigate whether, when EEG is feasible, hybrid systems could further improve behavioral outcomes beyond exclusive EEG- or EMG-based feedback. Ultimately, personalized training may require systematic characterization of many interacting factors, including measures of spasticity that could help to disambiguate muscle co-contraction and synergistic neural drive, for example, modified Ashworth scale, pathological synergies, muscle strength, attention, motivation, and magnetic resonance imaging scans to investigate the structural and functional integrity of distinct neural circuits.

While such a high degree of personalization is not yet practical, adaptive rehabilitation systems that can be self-administered and taken home are within reach. Our study demonstrates that relatively simple measures of muscle activation, in combination with commercial virtual reality equipment, may be sufficient to promote recovery even in very severely impaired individuals. Although utilization of muscle–computer interfaces for stroke rehabilitation is rare, it seems from our study that this method is not only feasible, but capable of producing neural changes and improvement on clinical evaluations. However, achieving significant outcomes may require interventions of a higher dose and with weekly behavioral measurements to capture the evolution of recovery. Our study, along with other recent efforts [24], supports the idea that, given the existence of myographic activity, muscle–computer interfaces are feasible and may be especially practical in terms of cost, simplicity, and eventual application outside of a clinical or laboratory setting.

## Figures and Tables

**Table 1:** Participant demographics and baseline evaluations. FMA-UE, Fugl–Meyer assessment for the upper extremity; MOCA, Montreal cognitive assessment.

Participant	Sex	Age	Onset (Months)	Paresis	FMA-UE	MOCA
1	Male	66	34	Left	19	23
2	Male	42	34	Right	22	17
3	Male	64	56	Left	14	22
4	Female	53	28	Left	20	22

**Table 2:** Statistical comparisons for clinical assessments. FMA-UE, Fugl–Meyer assessment for the upper extremity; ARAT, action research arm test; SIS, stroke impact scale.

Assessment	t	p	Pre	Post
**ARAT**	**2.61**	**0.079**	**5.75 (8.85)**	**7 (9.38)**
Extension	2.27	0.108	6.75 (7.81)	10.5 (8.19)
**FMA-UE**	**2.43**	**0.093**	**18.75 (3.40)**	**23.25 (4.19)**
Grip More Imp.	1.25	0.299	8.67 (6.99)	9.44 (5.78)
**SIS-16**	**5.67**	0.011 *	**58.5 (10.08)**	**62.75 (9.29)**

Group level paired *t*-test results for each assessment, as well as the mean (SD) values for each in sessions 1 and 10 (pre and post, respectively). Bold font represents nonsignificant trends (*p* < 0.1) and * represents significant changes (*p* < 0.05).

**Table 3:** Group level analysis of within-game performance and grip strength across training sessions. ER, extensor ratio.

Activity	t	p	Pre	Post
**ER**	**2.58**	**0.082**	**8.94 × 10** **^−16^ (1.65 × 10^−15^)**	**0.80 (0.62)**
Extensors	1.81	0.168	−1.99 × 10^−16^ (1.32 × 10^−15^)	1.41 (1.56)
Grip	1.53	0.224	7.67 (6.39)	10.13 (4)
Flexors	0.91	0.431	−4.71 × 10^−16^ (7.37 × 10^−16^)	0.51 (1.11)
Success	−0.40	0.719	57.29 (7.65)	55.83 (5.57)
Threshold	−0.03	0.981	36.82 (19.35)	36.44 (21.44)

Bold font represents nonsignificant trends (*p* < 0.1).

## Data Availability

Raw experimental data are available in the GitHub repository “npnl/REINVENT_data” [88].
